# Engineered bacteriophages as programmable biocontrol agents

**DOI:** 10.1016/j.copbio.2019.11.013

**Published:** 2020-02

**Authors:** Phil Huss, Srivatsan Raman

**Affiliations:** 1 Department of Biochemistry, University of Wisconsin-Madison, Madison, WI, United States; 2 Department of Bacteriology, University of Wisconsin-Madison, Madison, WI, United States

## Abstract

Bacteriophages (or ‘phages’) can be potent biocontrol agents but their potential has not been fully realized due to inherent limitations of natural phages. By leveraging new tools in synthetic biology, natural phages can be engineered to overcome these limitations to markedly improve their efficacy and programmability. Engineered phages can be used for targeted detection and removal of pathogens, *in situ* microbiome editing, gene delivery and programmable control of phage-bacterial interactions. In this mini review we examine different ways natural phages can be engineered as effective biocontrol agents through a design-build-test-learn platform and identify novel applications of engineered phages in food biotechnology.

## Highlights

• Engineered bacteriophages are promising tools for use in food biotechnology.
• Diverse natural bacteriophages can be leveraged by engineering for specificity and infectivity.
• Engineered bacteriophages are potent tools for pathogen biocontrol.
• Engineered bacteriophages can be used for targeted delivery vectors and pathogen detection.

## Graphical abstract

**Figure:**
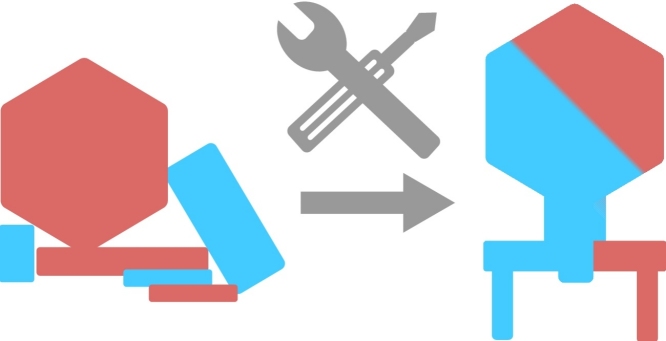

**Current Opinion in Biotechnology** 2020, **61**:116–121

This review comes from a themed issue on **Food biotechnology**

Edited by **Mark Blenner** and **Jan-Peter van Pijkeren**

For a complete overview see the Issue and the Editorial

Available online 17th December 2019

**https://doi.org/10.1016/j.copbio.2019.11.013**

0958-1669/© 2019 The Authors. Published by Elsevier Ltd. This is an open access article under the CC BY license (http://creativecommons.org/licenses/by/4.0/).

## Introduction

Foodborne infections caused by bacterial pathogens are a serious threat to human health with hundreds of thousands of deaths every year globally [1]. Healthcare costs associated with foodborne illness are estimated at a staggering $75 billion/year in the United States [2], with cascading economic losses from discarded food, culled farm animals, and food recalls. Traditional biocontrol of bacterial pathogens has relied on broad-spectrum approaches such as antibiotics or pasteurization that vary in effectiveness, impact natural microflora of food, and can negatively affect food quality [3,4]. Bacteriophages, or ‘phages’, are viruses that kill bacteria and are a promising alternative for bacterial biocontrol. They are ubiquitous natural predators of bacteria that are cheap to produce and can precisely target and kill pathogens without affecting food quality [5,6]. Although products based on natural phages have been in the market for decades, their adoption by industry is low and their game-changing potential remains unfulfilled [7]. Key factors impeding broader use of natural phages are poor efficacy compared to traditional biocontrol methods and poor scalability for high-volume production due to the narrow specificity profile of phages. However, synthetic biology offers exciting new tools to build engineered phages through a variety of recombineering approaches and *in vitro* genome assembly [8]. Engineering phages without natural limitations could lead to a design-build-test-learn platform to rapidly prototype new phages with user-defined properties. In this mini review, we will examine different ways natural phages can be engineered as more effective biocontrol agents and identify novel applications for engineered phages in food biotechnology.

## Engineering phages for higher efficacy

**Figure 1:**
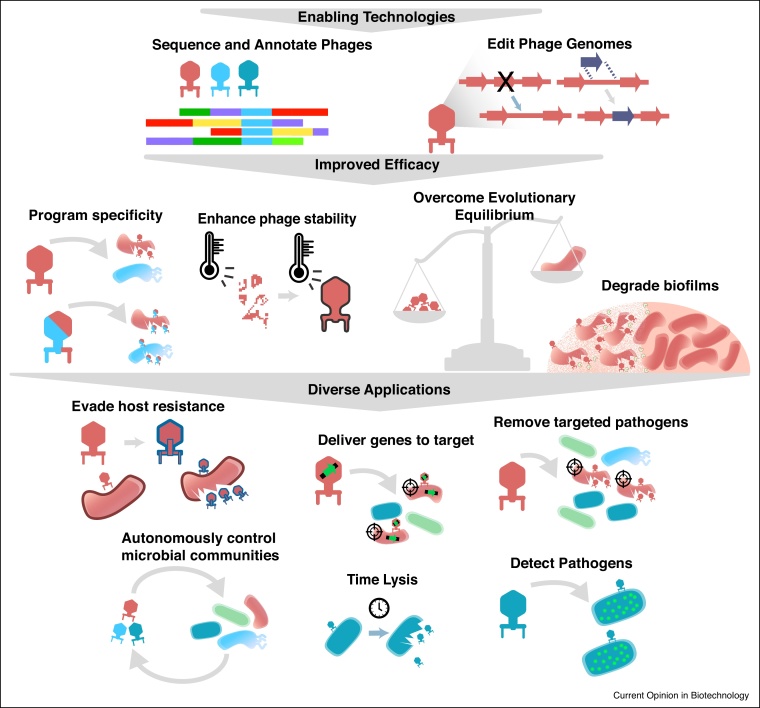
Strategies for engineering phages and their applications. The confluence of synthetic biology, genome engineering, viral metagenomics and deep sequencing has provided tools for rapid, evolution-guided and rational design of phages with tailored properties. Engineering approaches and applications in food biotechnology have been graphically summarized.

A major hurdle in the use of natural phages for biocontrol is their low efficacy. Although initial application of natural phages logarithmically reduces target bacterial levels, the residual bacterial load remains high. Even this limited efficacy is achieved with high phage to bacteria ratios which may not be feasible in applications outside a laboratory setting [9, 10, 11, 12, 13, 14]. Bacteria often continue to grow or quickly recover after phage application indicating low phage susceptibility and/or swift emergence of bacterial resistance, though propensity for resistance may be different in the wild [9, 10, 11,13, 14, 15, 16]. In this section, we evaluate factors that can limit the efficacy of natural phages and examine how engineering approaches can overcome these shortcomings (Figure 1).

### Overcoming evolutionary equilibrium in natural phages

Limited efficacy of natural phages is not surprising from an evolutionary perspective because a phage that fully eliminates its bacterial host will itself perish too. Therefore, as predators, phages must co-exist in equilibrium with their bacterial prey. In fact, evolutionary models show that natural selection favors mediocre killers over highly efficient phages [17]. Cocktails of several natural phages can be more efficacious but still face this evolutionary pressure to equilibrate with the bacterial host. To achieve a high pathogen clearance, we need to engineer phages which are not subject to evolutionary constraints imposed on natural phages. Every stage of a phage life cycle can be engineered to counteract various modes of resistance. For instance, phages with mutated tail fibers outcompete natural phages against *Pseudomonas aeruginosa* [18], and a single point mutation can confer a 1000-fold increase in efficacy for phages of *Mycobacterium abscessus* [19^••^]. Phage genome editing can remove built-in mechanisms in phages that reduce phage efficacy such as self-inhibition when their bacterial host experiences starvation [20,21]. Self-inhibition is beneficial for natural phages because it gives bacterial populations time to recover before phages prey again, but is undesirable for biocontrol. Engineered phages devoid of starvation-induced regulatory genes continued to grow on bacteria in starvation conditions [22]. Lytic capabilities of engineered phages have also been enhanced by overexpressing genes such as phage holins, which are proteins that permeabilize cell membranes [23]. Bacterial resistance can be overcome by incorporating new genes into the engineered phage genome based on specific mechanisms of bacterial resistance, for example phage defense mechanisms such as anti-CRISPR genes for bacteria that contain CRISPR or additional genes lethal to the target bacteria [24,25^••^,^26^].

A broad strategy to identify and overcome bacterial resistance is to employ genome-scale screens such as CRISPR interference or transposon insertion combined with phage replication to reveal mechanisms of resistance [27,28]. These screens are powerful tools that identify all host genes related to phage infection including genes responsible for bacterial resistance and genes utilized by the phage during infection. These screening tools can guide efforts to overcome evolutionary equilibrium. Expanding these screens to a larger set of bacteria could lead to understanding common resistance patterns and establishing engineering targets for phages.

### Engineering lysogenic to lytic conversion

Because of their high abundance, lysogenic phages represent a treasure trove of natural phages that can be used for biocontrol applications. During their lifecycle, lysogenic phages become dormant after integrating into the host genome, only to become activated later to kill the cell. Therefore, unlike lytic phages, lysogenic phages are not considered suitable for biocontrol and therapeutic applications due to limited efficacy and the risk of horizontal gene transfer. Natural phages must be thoroughly screened for lysogeny-related functions, significantly increasing time, cost, and uncertainty in product development cycles. To tap the vast natural phage reservoir, lysogenic phages could be converted to obligate lytic phages by removing factors that allow for lysogeny such as recombinases and repressors [29]. These converted phages have increased lethality and host range [25^••^,^30^], as evidenced by those used to treat *M. abscessus* showing a 10 000-fold increase in efficacy after removing lysogeny genes [19^••^]. New bioinformatics tools tailored to identify these lysogenic phages and the factors that make them lysogenic can quickly screen for candidate phages and genes for this conversion [31,32]. Lysogenic to lytic conversion greatly enhances the diversity and effectiveness of phages for biocontrol applications.

### Programming host specificity

The bacterial host range of different natural phages vary significantly and finding the right combination of natural phages can be arduous. Engineering phages to reprogram host range removes this constraint. Specificity can be tailored by swapping or complementing host-binding proteins [33,34,35^•^], or by removal of lysogeny genes or other genes that dictate specificity [30,36]. Programmable engineered phages would provide a platform for phage treatments for any of a set of closely related bacteria. Programming narrow host ranges is ideal for food reliant on specific microbiota compositions like cheeses where contaminating bacteria need to be removed without disturbing other microflora [37], whereas a programmable broad host range is more ideal for phages designed for biocontrol.

### Engineering stable phages

Phage stability is essential for biocontrol as phages are exposed to harsh environments including continual UV irradiation, low pH, and high temperature [9,38,39]. For example, pH and temperature in the gastrointestinal (GI) tract can vary between 2.5–5.7 and 38°C–42°C, respectively [40^•^]. Phages can degrade under these conditions, which can affect their proliferation and efficacy. Engineered phages with protective surface phospholipids have increased survival in the hostile GI tract environment [40^•^]. Some natural phages have also exhibited stability under harsh environmental conditions [41]. Genes responsible for these attributes can be identified and integrated into engineered phages to improve stability. Alternatively, approaches such as computational protein design, which has met with remarkable success in improving protein stability, could be employed to stabilize phage coat proteins without losing the flexibility required for function [42]. Stable phages could be used for crop protection and can also act as biocontrol ‘sentinels’ that can protect against bacterial contamination in the future.

### Degrading biofilms using engineered phages

Bacteria that form biofilms are an enormous challenge in food safety, as the biofilm provides a protective cover against traditional biocontrol agents including phages [43,44]. Phage enzymes that can degrade biofilms have been characterized [45] and phages engineered to include biofilm degrading peptides and enzymes have effectively dispersed biofilm [46^••^,^47^]. Phages targeting biofilm-creating organisms such as *Listeria monocytogenes, Staphylococcus aureus,* and *Escherichia coli* could be enhanced with these genes, establishing a unique advantage over traditional methods of biocontrol for these challenging pathogens.

## Other applications in food biotechnology

### Using engineered phages as targeted delivery vectors

Currently, we lack tools to deliver genes to specific targets in a mixed microbial community *in situ*. Engineered phages are ideally suited as targeted delivery vectors due to their tailored host specificity [35^•^]. Gene delivery is useful to modulate the composition of a community by enhancing or reducing the fitness of a target species. This approach can also deliver enzymes or metabolic operons to produce nutrients or signaling molecules. Engineered phages have been used as delivery vectors to resensitize bacteria to antibiotics by providing a drug-sensitizing DNA cassette [48]. CRISPR-Cas systems engineered into phages have also successfully disrupted virulence genes in bacteria [34]. These powerful tools could be leveraged to deliver and incorporate advantageous genes into the genome, such as proteases needed for food flavoring or acid production genes for fermentation.

### Engineered phages can compete with undesirable phages

Complex mixed communities of starter cultures used to make cheese, yogurt, and other fermented milk products are highly susceptible to phage infection. Economic losses from discarded production batches and sanitizing equipment can be significant. Current methods such as air flow control and strict sanitary conditions for controlling phages are expensive and not particularly effective [49]. Counterintuitively, phages themselves may be the solution to this issue. Phages naturally compete with other phages and have anti-phage genes that can block expression of phage genes, prevent infection, or compete for insertion sites in the bacterial genome [50]. Engineering lysogenic phages to encode these anti-phage genes while removing their own ability to propagate could protect starter cultures from unwanted phages.

### Using engineered phages as rapid detection tools

Rapid, low-cost detection of bacterial pathogens is critical for food safety. Methods such as antibody tagging are effective but are not cost effective, and culture-based mechanisms are laborious and time consuming. Engineered phages can deliver a bioluminescent reporter enzyme to readily detect pathogens [46^••^,^51^,^52^] as a rapid and accurate tool during food processing or in final products.

### Using engineered phages for controlled lysis

Timely lysis of starter cultures can be beneficial. For example, starter culture lysis is an important consideration in cheese maturation [53]. Lysogenic phages could be engineered to lyse the cell under an inducible condition such as access to a small molecule. Engineered lysogenic phages with this ability would trigger controlled, reproducible, and exponential lysis of the culture at an ideal time for cheese maturation.

## Platform for rapid phage engineering

When creating phage-based products, engineered phages have several key advantages over natural phages. Natural phage discovery is a serial, time consuming, and laborious process. The physiology of newly discovered phages is often poorly understood, which may lead to batch-to-batch inconsistencies during manufacturing and making mass production unsustainable. Natural phage production pipelines can also be interrupted by a frequent need to discover new natural phages to combat emerging bacterial resistance or to create cocktails to cover strain variations in pathogens.

**Figure 2:**
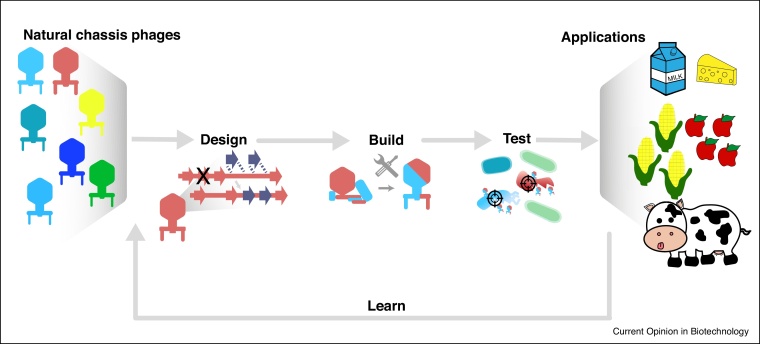
A platform for rapid phage engineering. A generalized platform for rapid engineering of phages using a design-test-build-learn approach.

Engineered phages could provide a flexible product development platform and a scalable and customizable workflow (Figure 2). We envision developing well-characterized chassis phages against different key bacterial clades and engineering them using natural and synthetic parts to achieve the desired bacterial host range and effect. Mixing and matching these chassis phages may require minor adjustments but not a complete overhaul of a production pipeline, minimizing batch-to-batch variability and ensuring product quality.

Regulatory considerations also play a role in development of phage-based products. Engineered phages and associated intellectual property can be patented and protected, but regulations for the use of these engineered phages are currently being developed by regulatory agencies [54]. Close cooperation with regulatory agencies to ensure compliance will be required to successfully develop engineered phage products.

## Conclusions

In this review we have outlined methods for improving natural phages for use in many applications in food biotechnology. Phages have enormous potential and are diverse tools that we have barely begun to explore. Phages are tractable and can be modified in many ways to improve effectiveness with even minimal engineering, and numerous methods now exist for modifying phage genomes to produce engineered phages. We envision that engineered phages will serve as a platform for developing biocontrol and delivery mechanisms to a broad range of bacteria to solve a variety of current problems in food biotechnology.

## Conflict of interest statement

S.R. is a member of the scientific advisory board of a phage therapeutic company MAP/PATH LLC. The authors declare no other conflict of interest.

## References and recommended reading

Papers of particular interest, published within the period of review, have been highlighted as:

• of special interest
•• of outstanding interest
